# Impact of anesthetic technique on troponin I levels in pediatric cardiac surgery: a randomized clinical trial

**DOI:** 10.1016/j.bjane.2025.844603

**Published:** 2025-03-01

**Authors:** João Victor Galvão Barelli, David D. Araujo, Suely P. Zeferino, Gustavo M. Dantas, Filomena B. Galas

**Affiliations:** a Faculdade de Medicina da Universidade de São Paulo, São Paulo, SP, Brazil; b Faculdade de Medicina da Universidade de São Paulo, Departamento de Anestesia, São Paulo, SP, Brazil

**Keywords:** Anesthesia, inhalation, Cardiac surgical procedures, Congenital heart defects, Extracorporeal circulation, Myocardial ischemia, Sevoflurane

## Abstract

**Background:**

This study aimed to evaluate the effects of the inhalational anesthetic sevoflurane on postoperative myocardial injury and renal function in children under 2 years old with congenital heart disease (RACHS 1, 2, and 3) undergoing cardiac surgery with extracorporeal circulation.

**Methods:**

A randomized clinical trial was conducted with 66 patients divided into two groups: one receiving sevoflurane and the other Total Intravenous Anesthesia (TIVA). The primary outcome was the serum troponin I levels within the first 48 hours postoperatively. Secondary outcomes included urine output and serum urea levels.

**Results:**

The median troponin I levels at 48 hours were 10.5 ng.mL−1 (IQR: 8.2–12.7) in the sevoflurane group and 11.0 ng.mL^−1^ (IQR: 8.7–13.0) in the TIVA group (p = 0.336). The sevoflurane group showed higher urine output on the second postoperative day (median: 800 mL [IQR: 420–913] vs. 541 mL [IQR: 312–718], p = 0.034) and lower serum urea levels (median: 24 mg.dL^−1^ [IQR: 16–35] vs. 36 mg.dL^−1^ [IQR: 23–49], p = 0.030).

**Conclusions:**

While sevoflurane did not significantly impact myocardial injury markers, it demonstrated potential renal protective effects in this patient population. Further research is necessary to confirm these findings across different pediatric age groups and surgical contexts.

## Introduction

The selection of the appropriate anesthetic technique is vital for optimizing outcomes in pediatric cardiac surgery. Hemodynamic stability and organ protection provided by specific anesthetics can significantly impact both intraoperative management and postoperative recovery.^1^ Inhalational anesthetics, particularly sevoflurane, have been extensively studied for their cardioprotective properties, especially regarding ischemia-reperfusion injury during Cardiopulmonary Bypass (CPB).^2^

The cardioprotective potential of halogenated anesthetics was first observed in experimental studies. Freedman et al. demonstrated that enflurane could improve functional recovery of the ischemic myocardium in isolated rat hearts, paving the way for clinical exploration of these effects.^3^ Subsequent research suggests that sevoflurane may induce preconditioning effects that increase myocardial tolerance to ischemic events, reducing perioperative myocardial injury.^4^^,^^5^ The underlying mechanism includes reducing myocardial oxygen consumption and stabilizing cellular membranes, limiting ischemia-reperfusion injury.^4^

The concept of Remote Ischemic Preconditioning (RIPC), introduced by Przyklenk et al. in 1993, advanced myocardial protection understanding. RIPC involves brief ischemic episodes in remote tissues, conditioning the heart to endure longer ischemic periods. This effect is mediated by intracellular signaling pathways, such as protein kinase activation and mitochondrial function modulation, which ultimately reduce cell death and preserve myocardial function.^6^^,^^7^

Symons and Myles’ meta-analysis of nearly 3,000 patients found that those anesthetized with volatile agents like sevoflurane had better cardiac outcomes, including reduced serum troponin I levels, shorter mechanical ventilation duration, and decreased hospital stays.^8^ Similarly, Landoni et al. reported that volatile anesthetics were associated with significantly reduced mortality and cardiac morbidity in adult cardiac surgery patients.^9^

Evidence for the benefits of anesthetic techniques in pediatric patients, especially those under two years old, remains sparse and inconsistent due to unique physiological challenges. These challenges include differences in myocardial metabolism, autonomic regulation, and immature enzymatic systems affecting drug pharmacodynamics.^10^^,^^11^ Additionally, the complexity of congenital heart diseases and surgeries in this population necessitates a highly individualized anesthesia approach.^12^

Elevated troponin I levels are strongly associated with adverse outcomes, including low cardiac output, prolonged hospital stays, and higher mortality.^13^^,^^14^

This study evaluated the impact of inhalational anesthesia with sevoflurane versus Total Intravenous Anesthesia (TIVA) on troponin I levels within 48 hours postoperatively in pediatric patients undergoing congenital heart defect surgery.^15^ Secondary objectives included evaluating other cardiac biomarkers and renal function parameters to determine sevoflurane's potential renal protection, particularly given CPB's nephrotoxicity.^16^ Additional outcomes, such as mechanical ventilation duration, ICU and hospital stay length, postoperative complications, and 30-day mortality rates, were also analyzed.

## Methods

### Study design

This study was a prospective, randomized, controlled trial conducted at the Heart Institute of the Hospital das Clínicas, Faculty of Medicine, University of São Paulo (Incor-HCFMUSP). The study was designed to assess the impact of anesthetic techniques on serum troponin I levels in pediatric patients undergoing corrective surgery for congenital heart defects. The protocol was approved by the Ethics Committee for Research Project Analysis (CAPPesq) under the number 513.854, and the study was registered by Filomena R B G Galas at ClinicalTrials.gov (Identifier: NCT03630796). Informed consent was obtained from the parents or legal guardians of all participants before inclusion in the study.

### Patients

Eligible patients were children up to 2 years old with congenital heart defects, classified as RACHS-1 categories 1, 2, or 3, who were scheduled for elective cardiac surgery with cardiopulmonary bypass. Exclusion criteria included contraindications to inhalational anesthetics, previous general anesthesia within the last 30 days, renal dysfunction, participation in another clinical trial, or refusal of the parents or guardians to participate.

### Eligibility criteria

The inclusion criteria required patients to be under 2 years of age, undergoing elective cardiac surgery with cardiopulmonary bypass to correct congenital defects, and classified with a surgical risk by RACHS-1 of -3 or lower. Patients were excluded if they had contraindications to inhalational anesthetics, had undergone general anesthesia within the last 30 days, had renal dysfunction, were participating in another study, or if their parents or guardians refused participation.

### Clinical investigation outline

Patients were randomly assigned to one of two groups: the Sevo group, which received balanced anesthesia with sevoflurane, or the TIVA group, which received total intravenous anesthesia. Patients were randomized 1:1 using computer-generated sequences. The randomization and group allocation were conducted immediately before the patient's admission to the operating room. A sealed envelope containing the group assignment was opened, and protocol instructions were provided to the anesthesiologist and perfusionist responsible for the case.

In the operating room, patients were monitored using continuous cardiac monitoring, pulse oximetry, and non-invasive blood pressure initially, followed by invasive blood pressure monitoring as per institutional protocols.

### TIVA group

Anesthesia induction was performed with ketamine (1‒3 mg.kg^−1^), midazolam (0.1‒0.5 mg.kg^−1^), fentanyl (2‒4 mcg.kg^−1^), and pancuronium (0.1 mg.kg^−1^), preceded by pre-oxygenation with an Inspired Oxygen Fraction (FiO2) of 40%‒100% and a fresh gas flow rate of 4‒8 liters.min^−1^. Anesthesia was maintained with additional doses of fentanyl (5‒20 mcg.kg^−1^) as required, along with continuous infusions of midazolam and ketamine at doses of 0.2‒0.8 mg.kg^−1^.hour^−1^ and 1‒2 mg.kg^−1^.hour^−1^, respectively, before and after cardiopulmonary bypass. During cardiopulmonary bypass, anesthetics were administered as needed, including fentanyl (1‒5 mcg.kg^−1^), midazolam (0.1‒0.5 mg.kg^−1^), and pancuronium (0.1 mg.kg^−1^).

### Sevo group

Anesthesia induction was achieved with sevoflurane, varying from 3%‒8%, with a fresh gas flow of 2‒10 L.min^−1^, in addition to fentanyl (2‒4 mcg.kg^−1^), pancuronium (0.1 mg.kg^−1^), midazolam (0.1‒0.5 mg.kg^−1^), and/or ketamine (1‒2 mg.kg^−1^) as per the anesthesiologist's discretion. In the Sevo group, anesthesia maintenance involved continuous administration of sevoflurane at 0.8–1.5 MAC, adjusted to the patients’ hemodynamic status. During Cardiopulmonary Bypass (CPB), sevoflurane was delivered through the oxygenator circuit to maintain consistent anesthetic depth. Supplemental intravenous agents, including fentanyl and midazolam, were used sparingly, as needed.

### Surgical and CPB management

All surgeries were performed by experienced pediatric cardiac surgeons using standardized techniques. CPB was conducted using a non-pulsatile flow, moderate hypothermia (28°‒32°C), and antegrade blood cardioplegia for myocardial protection. Blood product and vasoactive medication use was standardized in both groups to minimize variability.

### Outcomes

The primary outcome of the study was the serum troponin I curve measured at four time points: preoperatively, immediately postoperatively, at 24 hours postoperatively, and at 48 hours postoperatively. Secondary outcomes included the serum levels of CKMB, CPK, and BNP within the first 48 hours postoperatively, the incidence of postoperative complications such as renal dysfunction, need for dialysis, blood transfusion, or death, as well as the duration of mechanical ventilation, use of inotropic or vasopressor agents, and the length of stay in the ICU and the hospital.

### Statistical analysis

Based on previous studies^17^^,^^18^ and assuming a reduction of at least 2 ng.mL^−1^ in the primary outcome for the Sevo group compared to the TIVA group,^19^, ^20^, ^21^ with 80% statistical power and a 5% alpha error, the sample size was calculated to be 66 patients. The baseline characteristics, follow-up measures, and clinical outcomes were compared based on the intention-to-treat principle according to group allocation in a randomized study, as guided by the “Consort Statement”.^22^ Baseline characteristics, follow-up measures, and clinical outcomes were compared using the Student's *t*-test or Mann-Whitney *U* test for continuous variables and the Chi-Square or Fisher's exact test for categorical variables. Statistical significance was set at p < 0.05, and all tests were two-tailed. The analysis was performed using SPSS version 18.0 (SPSS Inc., Chicago, IL, USA).

Formal adjustments for multiple comparisons were not applied, as the analysis focused on a limited set of predefined outcomes with clear clinical relevance, minimizing the risk of type I errors. This approach ensures that the statistical interpretation remains aligned with the study's scope and objectives. Future research with larger sample sizes could incorporate comprehensive statistical adjustments to explore additional outcomes with greater precision.

## Results

### Patients

**Figure 1:**
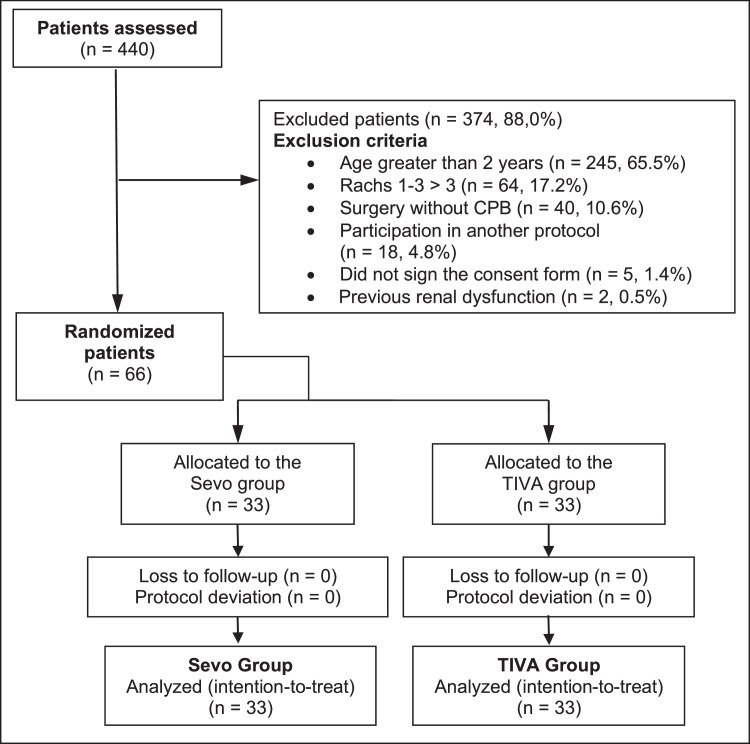
CONSORT flowchart. Source: Barelli, 2020.^23^ CPB, Cardiopulmonary Bypass; Rachs (Risk Adjustment for Congenital Heart Surgery): A scoring system used to stratify the complexity and risk of congenital heart surgery.

A total of 66 patients were included in the study, with 33 patients allocated to the Sevoflurane (Sevo) group and 33 to the Total Intravenous Anesthesia (TIVA) group. There were no protocol deviations in the Sevo group, whereas three patients in the TIVA group received Sevoflurane during surgery. Median age was 8 months (IQR: 5–12) in the Sevo group and 7 months (IQR: 5–10) in the TIVA group. Table 1 shows that gender distribution, weight, and pulmonary hypertension were comparable between groups, ensuring homogeneity. The ventricular septal defect correction was the most frequently performed surgery, performed in 7 patients (21.2%) in the Sevo group and 10 patients (30.3%) in the TIVA group, intraoperative characteristics of the patients can be checked on Table 2 (Fig. 1).

**Table 1:** Baseline and demographic characteristics of the patients.

Variable	Sevo Group (n = 33)	TIVA Group (n = 33)	p
Age (months), median and IQR	8 (5 – 12)	7 (5 – 10)	0.417^a^
Sex (Male)	15 (45.5%)	16 (48.5%)	0.805^b^
Weight (kg), median and IQR	6 (5 – 8)	6 (5 – 8)	0.797^a^
Height (cm), median and IQR	63 (57 – 69)	63 (59 – 71)	0.832^a^
Race			0.068^c^
White	28 (84.8%)	28 (84.8%)	
Black	0 (0%)	3 (9.1%)	
Mixed	3 (9.1%)	2 (6.1%)	
Oriental	2 (6.1%)	0 (0%)	
RACHS-1			0.831^c^
1	1 (3%)	2 (6.1%)	
2	15 (45.5%)	15 (45.5%)	
3	17 (51.5%)	16 (48.5%)	
Acyanotic Cardiopathy	25 (75.8%)	22 (66.7%)	0.415^b^
LVEF (%), median and IQR	72 (65 – 77)	73 (69 – 78)	0.318^a^
Right Ventricular Dysfunction	4 (12.9%)	4 (12.5%)	1.000^d^
Pulmonary Hypertension	13 (41.9%)	15 (48.4%)	0.610^b^
Previous Cardiac Surgery	3 (9.1%)	3 (9.7%)	1.000^d^

Source: Barelli, 2020.^23^

a Teste Mann-Whitney.

b Teste Qui-Quadrado.

c Teste razão de verossimilhança.

d Teste exato de Fisher. IIQ, Interquatile Interval; RACHS-1: Risk Adjustment in Congenital Heart Surgery; LVEF, Left Ventricular Ejection Fraction.

**Table 2:** Intraoperative characteristics of the patients.

Variable	Sevo Group (n = 33)	TIVA Group (n = 33)	p
Anesthesia time (min), median and IQR	445 (360 – 508)	455 (420 – 519)	0.284^a^
Surgery time (min), median and IQR	280 (235 – 318)	290 (258 – 340)	0.251^a^
CPB time (min), median and IQR	117 (85 – 155)	126 (91 – 157)	0.273^a^
Anoxia time (min), median and IQR	73 (51 – 116)	95 (67 – 111)	0.431^a^
Fluid balance (mL), median and IQR	7 (-265 – 103)	-56 (-212 – 134)	0.834^a^
Blood balance (mL), median and IQR	200 (114 – 256)	230 (150 – 341)	0.116^a^
Urine output (ml), median and IQR	310 (200 – 500)	400 (200 – 550)	0.438^a^
Crystalloid (mL), median and IQR	110 (93 – 195)	150 (100 – 275)	0.115^a^
Transfusion			
Red blood cells	26 (81.3%)	31 (96.9%)	0.104^b^
Plasma	19 (59.4%)	23 (71.9%)	0.292^c^
Platelets	6 (18.8%)	3 (9.4%)	0.474^b^
Cryoprecipitate	5 (15.6%)	4 (12.5%)	1^b^
Inotropes and vasopressors			
Dobutamine	11 (33.3%)	8 (24.2%)	0.415^c^
Milrinone	27 (81.8%)	28 (84.8%)	0.741^c^
Epinephrine	24 (72.7%)	25 (75.8%)	0.778^c^
Norepinephrine	1 (3.2%)	0 (0%)	1.000^b^
Nitric oxide	6 (18.2%)	4 (12.1%)	0.492^c^

Source: Barelli, 2020.^23^

a Mann-Whitney test

b Fisher's exact test.

c Chi-Square test. IQR, Interquartile Range; CPB, Cardiopulmonary Bypass.

### Myocardial ischemia markers

No significant differences were observed in myocardial ischemia markers. Median preoperative troponin I levels were similar: 0.05 ng.mL^−1^ (IQR: 0.01–0.10) in the Sevo group and 0.03 ng.mL^−1^ (IQR: 0.01–0.08; p = 0.336). Peak levels postoperatively reached 50 ng.mL^−1^ in both groups. CK-MB and CPK levels showed no significant differences between groups over the 48-hour postoperative period. Postoperative BNP levels were elevated in both groups without significant differences between Sevo and TIVA groups (Table 3).

**Table 3:** Myocardial ischemia markers and BNP in the first 48 hours postoperative.

Variable	Sevo Group (n = 33)	TIVA Group (n = 33)	p
**Troponin I (ng.mL^−1^)**			
T0 ‒ baseline	0.05 (0.01 - 0.1)	0.03 (0.01 – 0.08)	0.336^a^
T1 – POI	50 (50 – 114)	50 (50 ‒ 80)	0.554^a^
T2 – 1PO	33 (14 – 48)	29 (18 – 48)	0.834^a^
T3 – 2PO	12.34 (5.46 – 29.39)	16.35 (8.57 – 24.1)	0.510^a^
**CPK (U.L^−1^)**			
T0 – baseline	92 (58 – 122)	71 (55 ‒ 125)	0.347^a^
T1 – POI	1626 (920 – 2137)	1344 (938 – 2184)	0.803^a^
T2 – 1PO	1099 (673 – 1378)	958 (768 – 1496)	0.868^a^
T3 – 2PO	419 (254 – 650)	439 (286 – 1047)	0.826^a^
**CKMB (ng.mL^−1^)**			
T0 – baseline	3 (2 – 6)	3 (2 – 4)	0.482^a^
T1 – POI	171 (97 – 218)	142 (88 – 217)	0.756^a^
T2 – 1PO	55 (32 – 89)	81 (38 – 99)	0.279^a^
T3 – 2PO	14 (10 – 22)	20 (11 ‒ 34)	0.094^a^
**BNP (pg.mL^−1^)**			
T0 – baseline	70 (34.5 – 114.75)	103.5 (39 – 219.5)	0.222^a^
T1 – POI	103 (39.25 – 205.25)	149 (53 – 330)	0.222^a^
T2 – 1PO	629 (467 – 1151)	543 (420 – 1205)	0.613^a^
T3 – 2PO	450 (267 – 696)	463 (271 – 848)	0.975^a^

Source: Barelli, 2020.^23^

a Mann-Whitney test. PO, Postoperative; CPK, Creatine Phosphokinase; CKMB, Creatine Kinase-MB; BNP, B-type Natriuretic Peptide.

### Renal function markers

**Figure 2:**
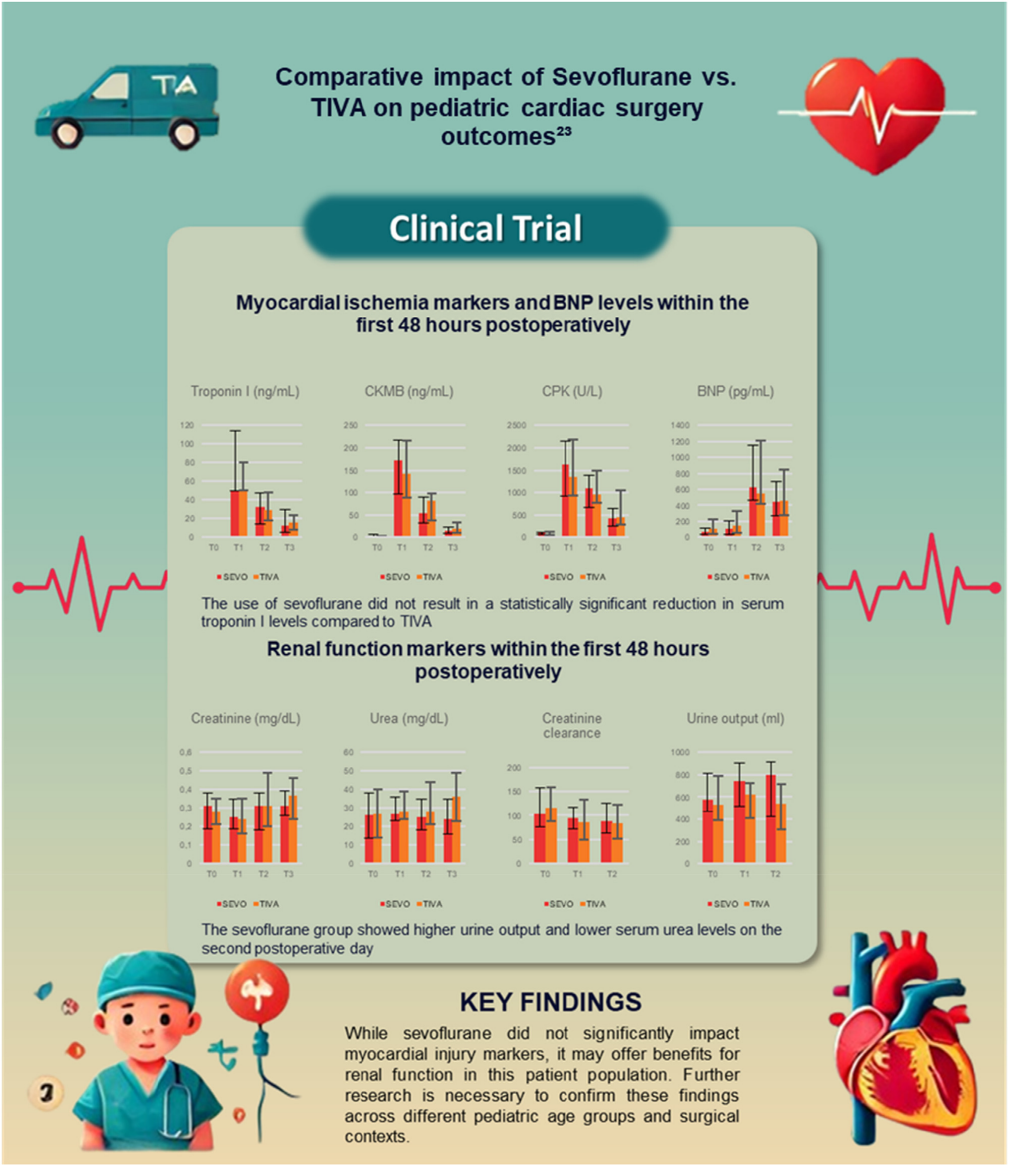
Comparative impact of Sevoflurane vs. TIVA on pediatric cardiac surgery outcomes.^23^ Source: Adapted from Barelli, 2020.^23^ This figure presents the comparison of myocardial ischemia markers (Troponin I, CKMB, CPK, and BNP) and renal function markers (Creatinine, Urea, Creatinine Clearance, and Urine Output) in pediatric patients undergoing cardiac surgery. The results indicate no statistically significant reduction in serum troponin I levels with sevoflurane compared to TIVA. However, the sevoflurane group exhibited higher urine output and lower serum urea levels on the second postoperative day, suggesting potential renal benefits.

On the second postoperative day, serum urea levels were higher in the TIVA group (36 mg.dL^−1^; IQR: 23–49) than in the Sevo group (24 mg.dL^−1^; IQR: 16–35; p = 0.030). Urine output was lower in the TIVA group on both the first and second postoperative days. Specifically, urine output on the first postoperative day was 625 mL (IQR: 406‒727) in the TIVA group compared to 741 mL (IQR: 520‒908) in the Sevo group (p = 0.031), and on the second postoperative day, it was 541 mL (IQR: 312‒718) in the TIVA group versus 800 mL (IQR: 420‒913) in the Sevo group (p = 0.034). Serum creatinine levels did not differ significantly between groups during the postoperative period (Table 4). Postoperative sedation protocols were standardized, utilizing midazolam and morphine infusions. Diuretics were administered as clinically indicated, with no significant differences in usage patterns between the groups. These measures ensured uniform postoperative care, supporting the interpretation of renal function outcomes (Fig. 2).

**Table 4:** Renal function markers in the first 48 hours postoperative.

Variable	Sevo Group (n = 33)	TIVA Group (n = 33)	p
**Creatinine (mg.dL^−1^)**			
T0 – baseline	0.31 (0.19 – 0.38)	0.28 (0.21 – 0.35)	0.753^a^
T1 – POI	0.25 (0.19 – 0.35)	0.24 (0.16 – 0.35)	0.761^a^
T2 – 1^st^ PO	0.31 (0.18 – 0.38)	0.31 (0.20 – 0.49)	0.264^a^
T3 – 2^nd^ PO	0.31 (0.26 – 0.39)	0.37 (0.24 – 0.46)	0.239^a^
**Urea (mg.dL^−1^)**			
T0 – baseline	26 ± 12	27 ± 13	0.831^a^
T1 – POI	27 (23 – 36)	28 (24 – 39)	0.443^a^
T2 – 1^st^ PO	25 (18 – 35)	28 (21 – 44)	0.459^a^
T3 – 2^nd^ PO	24 (16 – 35)	36 (23 – 49)	0.030^a^
**Creatinine clearance (mL.min.1.73 m^2^)**			
T0 ‒ baseline	104 (77 – 157)	116 (87 – 158)	0.631^a^
2^nd^ PO	94 (73 – 117)	85 (49 – 133)	0.324^a^
3^rd^ PO	89 (63 – 126)	84 (52 ‒ 122)	0.706^a^
**Urine output (mL)**			
T1 ‒ POI	572 (466 – 816)	530 (390 – 793)	0.457^a^
T2 – 1^st^ PO	741 (520 ‒ 908)	625 (406 – 727)	0.031^a^
T3 – 2^nd^ PO	800 (420 – 913)	541 (312 – 718)	0.034^a^

Source: Barelli, 2020.^23^

a Mann-Whitney test. POI, Immediate Postoperative; PO, Postoperative.

### Clinical outcomes

The majority of patients in both groups required blood transfusions, and the use of inotropic agents such as milrinone was consistent across both groups. The overall postoperative mortality was low, with no significant difference between the groups, indicating that both anesthetic techniques were similarly effective in this patient population (Table 5).

**Table 5:** Secondary outcomes between patients in the Sevo and TIVA groups.

Variable	Sevo Group (n = 33)	TIVA Group (n = 33)	P
Low Cardiac Output	5 (15.2%)	9 (27.3%)	0.228^a^
Cardiogenic Shock	3 (9.1%)	6 (18.2%)	0.475^b^
Arrhythmia	3 (9.1%)	4 (12.1%)	1.000^b^
Need for Ventricular Assist Device	2 (6.1%)	4 (12.1%)	0.672^b^
Combined Outcome	17 (53.1%)	17 (51.5%)	0.897^a^
Myocardial Ischemia	16 (50%)	13 (40.6%)	0.451^a^
Renal Dysfunction (Injury/Failure)	5 (15.2%)	8 (24.2%)	0.353^a^
Mechanical Ventilation Time (min), median and IQR	2310 (857 – 8812)	4500 (1162 – 8168)	0.434^c^
Inotrope Duration (min), median and IQR	6450 (2880 – 20160)	6205 (2827 – 11685)	0.629^c^
Vasopressor Duration (min), median and IQR	3945 (1124 – 15450)	4113 (1650 – 12555)	0.983^c^
ICU Stay (days), median and IQR	7 (4 – 16)	7 (4 – 12)	0.979^c^
Hospital Stay (days), median and IQR	20 (10 – 29)	16 (13 – 38)	0.695^c^
ICU Readmission	1 (3%)	0 (0%)	1.000^b^
Death	1 (3%)	4 (12.1%)	0.355^b^

Source: Barelli, 2020.^23^

a Chi-Square test.

b Fisher's exact test.

c Mann-Whitney test VM, Mechanical Ventilation; IQR, Interquartile Range.

## Discussion

This study evaluated the impact of the inhalational anesthetic sevoflurane on postoperative myocardial injury in pediatric patients undergoing cardiac surgery with Cardiopulmonary Bypass (CPB). Its rationale stemmed from evidence of sevoflurane's cardioprotective effects in adults, where it can reduce myocardial injury and improve outcomes.^15^^,^^19^ However, the extent to which these benefits apply to pediatric patients, who have distinct physiological characteristics and face unique surgical challenges, remained uncertain.

A Randomized Controlled Trial (RCT) design was used, ensuring that the sevoflurane and Total Intravenous Anesthesia (TIVA) groups were comparable at baseline. Such a design minimizes bias and strengthens the attribution of any observed differences in postoperative outcomes to the anesthetic technique rather than to confounding factors.^24^

The primary finding was that there was no statistically significant difference in postoperative troponin I levels between the sevoflurane and TIVA groups. Troponin I is a well-established biomarker for assessing myocardial injury after surgery.^25^ The absence of a significant difference (p = 0.336) questions the hypothesis that sevoflurane offers superior myocardial protection in pediatric patients. Unlike adult myocardium, the pediatric heart has higher metabolic rates, reduced contractile protein maturity, and different enzymatic activity. Such developmental factors may impair the cardioprotective mechanisms of sevoflurane, such as ischemic preconditioning. Furthermore, the inflammatory response and hemodynamic instability in pediatric CPB may mask potential anesthetic benefits. Thus, the cardioprotective effects documented in adults may not translate directly to younger patients due to fundamental differences in myocardial physiology and the unique stressors of pediatric cardiac surgery.

The findings underscore the importance of developmental factors. The pediatric heart undergoes significant changes that may influence its response to ischemia and anesthetic agents. Moreover, the complexity of congenital heart defects and the technical challenges of pediatric CPB may modulate the impact of any putative cardioprotective intervention, explaining why sevoflurane's benefits observed in adults were not replicated here.

Although myocardial protection was absent, the study identified potential renal benefits with sevoflurane. The sevoflurane group exhibited higher urine output and lower serum urea levels than the TIVA group, suggesting a renal protective effect during pediatric cardiac surgery. This finding is clinically relevant given the susceptibility of pediatric patients to renal complications.^26^ Randomization ensured balanced group allocation regarding surgical timing, and both groups followed similar postoperative protocols. Thus, differences in urine output and urea likely reflect the pharmacological effects of sevoflurane. The significant renal outcomes, such as for urea (p = 0.030), highlight their clinical relevance.

The renal protection associated with sevoflurane may have multiple underlying mechanisms. Sevoflurane may enhance renal perfusion by adjusting vascular tone, improving oxygen delivery during hemodynamic stress. Sevoflurane might reduce pro-inflammatory cytokines like IL-6 and TNF-alpha, which are commonly elevated during CPB. These mechanisms may collectively reduce renal injury related to CPB-induced hemodynamic changes and inflammation.

Renal protection is significant in pediatric CPB patients, who are prone to dysfunction from hemodynamic instability and inflammation. Renal injury can prolong hospital stays, increase morbidity, and lead to long-term consequences. Thus, sevoflurane's ability to enhance urine output and reduce serum urea could translate into tangible clinical benefits. While the study demonstrated statistically significant renal protection, additional research is needed to ascertain whether these short-term improvements lead to better long-term renal outcomes. Understanding these long-term implications is critical for optimizing perioperative management in pediatric cardiac surgery.

From a clinical perspective, the potential renal protection conferred by sevoflurane is noteworthy. Given the high incidence of renal dysfunction in this population and the long-term consequences that can arise from perioperative renal injury,^27^^,^^28^ a strategy that helps preserve renal function could be advantageous. Pediatric patients undergoing CPB are vulnerable to acute kidney injury, which often leads to prolonged hospitalization and increased morbidity. By improving renal parameters, sevoflurane might help reduce these risks and improve both immediate and extended outcomes. Although this study focused primarily on myocardial injury and renal function, evaluating inflammatory markers such as IL-6 and TNF-alpha in future research could clarify the anti-inflammatory role of sevoflurane and its influence on end-organ protection. Long-term renal follow-up studies would also help determine if these renal benefits persist and yield meaningful clinical improvements.

The clinical implications are multifaceted. While sevoflurane did not demonstrate the expected myocardial protection in this pediatric cohort, the potential renal benefits suggest that it may still play a valuable role in anesthetic management. Given the high incidence of renal dysfunction and its consequences in pediatric cardiac surgery, a strategy that helps preserve renal function could be advantageous. Even if the myocardial protective effects are less pronounced in pediatric patients, the renal protection alone may justify considering sevoflurane's use.

However, several limitations must be acknowledged. The rotation of clinical teams providing anesthesia and surgery according to the day and time of operation introduces potential variability.^29^ Although standardized protocols were followed, differences in team experience, technique, and intraoperative decision-making may have influenced outcomes. Moreover, this study focused on children under two years of age with RACHS (Risk Adjustment for Congenital Heart Surgery) categories 1–3, limiting its generalizability. Future studies should examine broader age ranges, more complex congenital heart defects, and different RACHS categories. Such investigations would help confirm these findings and determine whether the observed effects are consistent across diverse patient populations.

Additional studies are required to clarify the role of inhalational anesthetics in pediatric patients. Future trials should systematically evaluate sevoflurane across a range of congenital heart defects, different age groups, and surgical techniques. Considering additional endpoints, such as inflammatory biomarkers, would provide a more comprehensive understanding of sevoflurane's impact. Larger, standardized studies with consistent team involvement and defined anesthetic protocols could more accurately assess the myocardial and renal effects of sevoflurane, potentially leading to refined anesthetic strategies in pediatric cardiac surgery.^30^

Overall, this study did not find sevoflurane superior to TIVA in reducing postoperative myocardial injury in pediatric patients undergoing CPB. The anticipated cardioprotective effects observed in adults were not replicated, possibly due to developmental differences in myocardial physiology and the distinct challenges of pediatric cardiac surgery. Nevertheless, the renal benefits associated with sevoflurane were significant and suggest that it may still provide meaningful clinical advantages. This finding highlights the complexity of anesthetic management in pediatric cardiac surgery and the need for tailored approaches considering both cardiac and non-cardiac outcomes. While further investigation is essential, the renal protection demonstrated by sevoflurane indicates its potential role in improving overall perioperative management and postoperative outcomes in this vulnerable population.

## Conclusion

In children under 2 years with congenital heart disease (RACHS 1, 2, and 3) undergoing cardiac surgery with extracorporeal circulation, the use of the inhalational anesthetic sevoflurane did not significantly reduce serum troponin I levels within the first 48 hours postoperatively compared to total intravenous anesthesia. However, sevoflurane was associated with improved renal function parameters, suggesting potential renal protective effects in this population. Further studies are needed to define the optimal role of inhalational anesthetics in pediatric cardiac surgery.

## Institutional Research Board Approval

Approval Number: 513.854

Date of Approval: January 22, 2014

Registry of the Study:

Registration Number: NCT03630796

Date of Registration: August 20, 2018

## Prior Presentations

Not applicable.

## Funding

Support was provided solely from institutional and/or departmental sources.

## Conflicts of interest

The authors declare no have conflicts of interest.
